# Quality assurance: Fundamental reproducibility tests for 3D treatment‐planning systems

**DOI:** 10.1120/jacmp.v6i3.1983

**Published:** 2005-08-17

**Authors:** Charles M. Able, Michael D. Thomas

**Affiliations:** ^1^ Department of Radiation Oncology High Point Regional Cancer Center, Wake Forest University School of Medicine, Medical Center Boulevard Winston‐Salem North Carolina 27157 U.S.A.

**Keywords:** AAPM TG‐53, ADAC Pinnacle^3^ QA, quality assurance

## Abstract

The use of image‐based 3D treatment planning has significantly increased the complexity of commercially available treatment‐planning systems (TPSs). Medical physicists have traditionally focused their efforts on understanding the calculation algorithm; this is no longer possible. A quality assurance (QA) program for our 3D treatment‐planning system (ADAC Pinnacle^3^) is presented. The program is consistent with the American Association of Physicists in Medicine Task Group 53 guidelines and balances the cost‐versus‐benefit equation confronted by the clinical physicist in a community cancer center environment. Fundamental reproducibility tests are presented as required for a community cancer center environment using conventional and 3D treatment planning. A series of nondosimetric tests, including digitizer accuracy, image acquisition and display, and hardcopy output, is presented. Dosimetric tests include verification of monitor units (MUs), standard isodoses, and clinical cases. The tests are outlined for the Pinnacle^3^ TPS but can be generalized to any TPS currently in use. The program tested accuracy and constancy through several hardware and software upgrades to our TPS. This paper gives valuable guidance and insight to other physicists attempting to approach TPS QA at fundamental and practical levels.

PACS numbers: 87.53.Tf, 87.53.Xd

## I. INTRODUCTION

In recent years, with the advent of image‐based 3D treatment planning, commercially available treatment‐planning systems (TPSs) have significantly increased in their level of complexity. Historically, the clinical medical physicist could focus on understanding the intricacies of the calculation algorithm and have a cursory knowledge of peripheral devices and systems. This is no longer possible. Image‐based TPSs must now interface with various imaging systems and other informatic systems that control the delivery device and are typically a subnet of the institution's computer network. Increasingly, treatment approaches are being determined by the limits of the delivery device as modeled using the TPS.^(^ ^1^ ^)^ The resulting treatment plan is not always intuitive; therefore, a high level of confidence in the final output must be insured at the time of implementation and maintained through a comprehensive quality assurance (QA) program.

The process of radiation therapy is well known but complex. There are a number of procedural steps. An integral step is the treatment‐planning process. There are a number of documents^(^ ^1^ ^–^ ^4^ ^)^ that discuss QA for the entire treatment‐planning process as well as treatment‐planning computers. Most recently, the American Association of Physicists in Medicine published its first guidelines on the topic of treatment‐planning quality assurance—Radiation Therapy Committee Task Group 53: Quality assurance for clinical radiotherapy treatment planning.^(^ ^1^ ^)^

The TG‐53 document provides general guidance on a wide range of tests for the treatment‐planning process. It gives valuable insight into the need for and inclusion of various tests in all aspects of treatment planning. It is left to the medical physicist to make decisions concerning the structure of the QA program at his or her facility. Currently, there are very few documents that detail specific tests that are appropriate to specific TPSs in specific clinical environments. This level of detailed information may assist the clinical physicist to more rapidly institute vital QA and reduce the time required in program development. This program has been implemented/tested in the clinic, found useful, and ascribes to TG‐53 guidance.

Quality assurance guidance documents^(^ ^1^ ^,^ ^2^ ^)^ and related articles^(^ ^3^ ^–^ ^5^ ^)^ focus on QA of the radiation treatment‐planning process, which includes the following:

- patient positioning/immobilization
- image acquisition
- anatomy definition
- beam/source technique
- dose calculations
- plan evaluation
- plan implementation
- plan review

Using this approach, QA can be implemented in a comprehensive manner. Generally speaking, community cancer centers incorporate some but not all of these components into their QA programs. Emphasis is usually placed on those aspects deemed “critical” to the process. In fact, any aspect of the process not included in the QA program can result in failure of the process. The physicist must determine the balance between cost and benefit for various process steps.

When developing QA for the TPS, the QA components that must be addressed are the following:

1. acceptance testing
2. commissioning
3. routine QA (reproducibility) testing

Acceptance testing confirms that the TPS performs according to its manufacturer's or institution's specifications. Commissioning determines the accuracy of the TPS under various performance conditions. Reproducibility testing ensures constancy of operation and the results produced by the TPS.

While the clinical physicist is professionally charged with promulgating specifications and acceptance tests for the new TPS to be acquired, in practice, this is seldom the case. An honest and pragmatic description of a new TPS acquisition would reveal that the physicist understands the underlying algorithms used, the hardware capabilities of the computer system, and the mechanics of treatment planning as implemented by the vendor, but seldom has the understanding of and experience with the vendor's radiotherapy planning system process to perform more than the vendor provided acceptance testing. This vendor‐provided acceptance testing seldom demonstrates more than the functionality of the system from a clinical medical physics point of view. Instead, much of the acceptance testing of the software is shifted to the commissioning process. During the commissioning process, appropriate analysis of the TPS results is facilitated when the physicist has completed system training and expanded his or her knowledge base of the TPS.

The TPS commissioning process has become an evolutionary process due to the complexity of the systems and the way in which the systems are used in a given department.^(^ ^2^ ^,^ ^3^ ^)^ Multiple pathways for generating a given treatment plan are a feature that makes the modern TPS such a powerful tool. They also make exhaustive commissioning virtually impossible in a clinical environment. Therefore, the TPS is commissioned for the way it will be used in the specific clinical setting, and as the need for additional features evolves, these components are commissioned.

The commissioning process as described in TG‐53 includes two distinct components: nondosimetric testing and dosimetric testing. Nondosimetric aspects are those not directly related to dose calculation. These include but are not limited to proper calibration and operation of peripheral devices (digitizers, film scanners, printers, etc.), proper calibration and transfer of data from networked imaging systems (CT, MRI, etc.), proper handling of anatomical structures and reference definitions (2D and 3D structures, regions of interest), beam positioning and definition, and hardcopy output format and accuracy. The dosimetric testing includes a wide range of tests depending on the features being used. External beam treatment planning includes verification of the accuracy and self‐consistency of the input dataset, proper format and accuracy of data input to the system, relative dose calculation verification (comparing measured and calculated dose), absolute dose output and plan normalization, and clinical test case verification.

Routine QA testing or reproducibility testing is essential for maintaining a high level of confidence in the integrity of treatment‐planning results. The reproducibility tests, as they will be referred to in this document, are typically a subset of the commissioning tests. Reproducibility testing includes nondosimetric and dosimetric components.

The focus of this paper is external photon beam treatment‐planning QA for the Philips ADAC Pinnacle^3^. Fundamental reproducibility tests are presented as required for a community cancer center environment using conventional and 3D treatment planning. A comprehensive QA program for external beam treatment planning is not presented. The authors propose an approach that is consistent with the format presented in TG‐53 and balances the cost versus‐benefit‐equation confronted by the clinical physicist in a community cancer center environment. The tests presented are narrow in scope and represent testing that may be characterized as idiosyncratic to the current evolution of treatment planning at our facility. We believe this will give some valuable guidance and insight to other physicists attempting to approach TPS QA at fundamental and practical levels.

## II. METHODS AND MATERIALS

### A. Facility background and system use

The Phillips Cancer Pavilion at High Point Regional Health System in High Point, North Carolina, is a radiation oncology department in transition from conventional radiotherapy to intensity‐modulated radiotherapy (IMRT). The department treats approximately 40 to 45 patients per day with a Varian 2100 SCX and a Varian Clinac 2100C LINAC. In the past 24 months, the department has fully implemented 3D treatment planning, upgraded the Varis Record and Verify system from version 1.4 to generation 6, introduced digital imaging via the Kodak ACR 2000i computed radiography system (portal imaging), and installed Ximavision software on the Ximatron Simulator and the VARIS Vision mini‐PACS system. Our patients are imaged using conventional CT scanners in the radiology department. The scans are transferred electronically to the TPS.

The Philips ADAC Pinnacle^3^ system (using a single Ultra 2 workstation) was fully implemented for external photon beam treatment planning in the spring of 2001. This system was upgraded in December 2002 to two fully functional workstations (SunBlade) and two PinnacleMD workstations for physician use in anatomical structure delineation and plan review. The only use for this TPS is external photon beam (forward) treatment planning. There is no electron beam or brachytherapy planning. The scope of the reproducibility testing presented is indicative of the level of current utilization of external beam treatment planning at High Point Regional.

### B. Nondosimetric QA tests

Treatment planning includes the use of the simulator for patient positioning and/or immobilization for all patients. Approximately 20% of our patients are simulated in a conventional manner (film and manual contour), and the remaining 80% receive a virtual simulation based on CT scans alone or CT and MRI fusion. A subsequent verification (radiographic/fluoroscopic) simulation is performed on all patients receiving virtual simulation. The nondosimetric testing we performed is done to ensure accurate format and transfer of TPS input and output information. The integrity of the external coordinate system of the simulator and CT scanners and their transfer to the patient coordinate system by the TPS are important for accurate treatment delivery. Nondosimetric testing on the electromagnetic digitizer, CT scanner data, and hardcopy output (Table 1) are performed to ensure scan fidelity and correct coordinate transformation.

**Table 1:** Quality assurance program

	**Three‐dimensional treatment planning system**		
	**Nondosimetric tests**		
Test	Description	Tolerance	Periodicity
electromagnetic digitizer accuracy	verification of accurate digitizer input and output of a 40 cm×40 cm contour and various calculations points	±1 mm	quarterly
image acquisition, use, and display	review performance characteristics following system calibration by service representative. Scan electron density phantom per protocol and review results at scanner console and after transfer to TPS	Water $0\pm 10)\text{Air‐}1000\pm 10{\text{CT vs Pinnacle}}^{3}\pm 15$ measurements ±1% of expected value	quarterly semi‐annually
ROI, margin generation, and DVH accuracy	Verification of the constancy of display and determination of known ROIs, DVHs, and automatic generation of structure margins (voxel size 2)mm×2)mm×2)mm)	3% or 1 cm^3^, whichever is smaller	semi‐annually
hardcopy output verification	review and verification of text printout, 2D irregular field output, and 2D isodose output for proper format relative to baseline and accuracy of parameters and position of points and isodose lines	must be identical to baseline and input data	semi‐annually
Dosimetric tests			
Test	Description	Tolerance	Periodicity
monitor unit verification	monitor unit calculation constancy check of various field sizes, depths, and SSDs (open and wedges) for all energies	0.5% or 1 MU, whichever is greater	semi‐annually
standard isodose verification	square field isodose constancy check (on‐and off‐axis)	• <0.5 cm on cental axis • 1 mm in high‐dose gradient regions	semi‐annually
clinical case verification	isodose and monitor unit constancy check of several clinical cases (i.e., CT plans of prostate, breast, etc.; mantle irregular field)	• monitor units must be identical to baseline data • visual evaluation relative to baseline isodoses	semi‐annually

The electromagnetic digitizer accuracy tests consist of the entry of a simple manual contour with various dose points. The coordinate accuracy is verified using mouse/cursor readout and a review of the documented coordinates of known points on the hardcopy printout. The standard contour is a 40 cm×40 cm square with dose points located at the center of each quadrant. This test ensures that over 90% of the usable surface of the digitizer is tested for positional and geometrical accuracy. The maximum expected deviation is 1 mm.

The CT scanner is tested for proper image acquisition, use, and display. This type of testing has been well established.^(^ ^1^ ^–^ ^6^ ^–^ ^10^ ^)^ The approach used educates CT service personnel and CT departmental staff on how radiation therapy uses the information obtained from patient scans. The increased awareness has generated a greater level of cooperation and coordination.

An electron density phantom is scanned per QA protocol (Table 1). The QA protocol requires the CT staff to scan the phantom and (1) to evaluate the mean CT number for each insert (lung, water, bone, muscle, etc.) using a region of interest (ROI) equivalent to 100 pixels, and (2) to measure the distance between two known points. The original unmodified scans are transferred to the TPS electronically, and a hardcopy of the CT staff evaluation is sent to radiation therapy. The transferred images are subjected to the following tests:

- grayscale window and level settings
- geometrical accuracy of slices associated with images
- ROI analysis
- positional measurements
- image fidelity

The images are evaluated for CT number (Hounsfield unit) accuracy on the TPS in the same manner using the ROI tool (statistics tab). A distance measurement is performed and compared to the CT scan result. Verification of the aspect ratio is performed. Table 1 contains the maximum acceptable deviation or variability of CT QA results.^(^ ^6^ ^)^

Baseline QA data for these tests was much more extensive and verified constancy of CT number versus slice thickness, field of view size, and scan position within scan. Baseline measurements are repeated during preventative maintenance. A copy of the preventative maintenance results is then forwarded to radiation oncology for review and is part of our QA record.

Evaluation of the accuracy of ROI determination (area and volume), automatic margin generation, and dose‐volume histogram (DVH) calculation was performed using a commercially available phantom/system.^(^ ^11^ ^)^ Using this system, objects of known size, orientation, and geometrical shape are contoured and evaluated using the TPS's measurement and evaluation tools. The TPS results are compared to those provided by the manufacturer. A similar method is used to measure the volume of a given object enclosed by a specific isodose line. The measured volume is compared to the volume calculated by the DVH tool provided. These evaluations are critical to accurate utilization of the 3D dataset.^(^ ^12^ ^)^

Hardcopy output accuracy is essential to the proper documentation and interpretation of the treatment delivered. In addition, the hardcopy format must be checked for constancy. In the event of a software change, the change in format must be approved prior to clinical use. Baseline hardcopy printouts of irregular field plots, 2D isodose plots, and text printout of machine/energy, setup source to surface distance (SSD), beam orientation, etc., are compared using hardcopy from the digitizer and dosimetric tests presented in the next section.

### C. Dosimetric QA test

Dosimetric QA is limited to three distinct types of testing: monitor unit (MU) calculation accuracy (absolute dosimetry), isodose constancy (relative dosimetry), and clinical case evaluation (isodose and monitor unit constancy) (Table 1).

Monitor unit accuracy calculations are performed under the following conditions. A 50 cm(W)×40 cm(L)×30(H) homogeneous water phantom is entered in the TPS clinical workspace. This is accomplished using a manual contour entry with each slice spaced 0.5 cm. The calculation grid covered the entire volume with a resolution (voxel size) of 0.4 cm×0.4 cm×0.4 cm. Reference points were entered, along the central axis on the central slice, every centimeter from 0 cm to 20 cm. In addition, a reference point at the reference depth for each energy to be tested (i.e., 6 MV=1.5 cm;15 MV=2.7 cm) was entered. For each field size, energy and SSD the beam was normalized separately to the reference depth, 5 cm and 20 cm using individual prescriptions, each delivering 100 cGy to its specified point. This was done for SSDs of 90 cm, 100 cm, and 110 cm. Table 2 summarizes the wide range of MU calculations performed. While these calculations are done in standard geometry, they represent the relevant clinical range of use of the system. The ability of the system to normalize the dose to a given point, correct for changes in SSD, and apply the correct prescription to a given beam and given point is tested for a variety of conditions and beam modifiers.

**Table 2:** Monitor unit verification

Machine‐Energy	Field size (cm)	SSD (cm)	Depth (cm)	Wedge angle
CL 21SCX–6 MV	5×5	90	$D_{\text{max}}$	Open
CL 21SCX–15 MV	10×10	100	5.0	15
	20×20	110	20.0	30
CL 2100c–6 MV				45
CL 2100c–15 MV				60

This approach tests a range of calculation pathways and the algorithm at its clinical limits, in terms of the data used to generate the model. Reproducibility testing of MU calculations over a range of clinically relevant conditions should identify changes to the dataset, corruption of the dataset, or small changes in the modifying terms of the beam model. All MUs must be exactly the same as baseline data results.

Verification of accurate isodose calculation and display is a primary task during the commissioning process.^(^ ^8^ ^)^ Direct comparison to measured isodoses can be accomplished using commercially available automated beam scanning systems. There are several methods that can be used to evaluate the comparison between measured and calculated isodose. The overlay method is used in this work. Measured isodoses are plotted, at the correct magnification, on acetate (transparency film). These are compared to the isodoses generated by the beam model during the TPS commissioning process. When the beam model is approved, the baseline isodoses for the reproducibility test are generated from the TPS and plotted on acetate.

Isodose constancy calculations are performed under the same conditions as the MU accuracy test. All calculations are normalized to the reference depth at 100 cm SSD. The acceptability criterion for isodose constancy is <0.5 mm along the central axis (low‐dose gradient region) and 1 mm in the penumbra region (high‐dose gradient region). Table 3 summarizes the isodose constancy tests performed. We chose to limit our isodose constancy to open field isodoses. Wedge isodoses were not included in an effort to balance the effort required versus what tests are necessary.

**Table 3:** Standard isodose verification

Field size (cm)	Plane	Position
5×5	cross plane	central axis
5×5	in‐plane	central axis
10×10	cross plane	central axis
10×10	in‐plane	central axis
20×20	cross plane	central axis
20×20	cross plane	+5.0 cm (toward gantry)
20×20	cross plane	—5.0 cm (toward table)
20×20	in‐plane	central axis
20×20	in‐plane	—5.0 (left facing gantry)
20×20	in‐plane	+5.0 cm (right facing gantry)

Machine‐Energy: CL 21SCX–6 MV; CL 21SCX–15 MV; CL 2100c–6 MV; CL2100c–15 MV

Setup: gantry angle=180° (down); collimator angle=180° (neutral)

Open fields at 100 cm SSD

Clinical test cases should be representative of the types of cases clinically relevant for the given facility. As mentioned previously, our facility is in transition from conventional radiotherapy to IMRT; thus test cases chosen reflect this state of transition: irregular field (mantle), breast tangents (multiple manual contour), and prostate (CT images). The test cases will evolve as new treatment techniques and methodologies are fully established in the clinic. Table 4 summarizes the test cases evaluated.

**Table 4:** Clinical case verification

Description	Field size (cm)	Gantry angles (°)	Points	Depth (cm)
AP mantle field	x1=10.0	180	central axis	reference depth
	x2=10.0			exit
CL 2100c–6 MV	y1=11.0			
CL 2100c–15 MV	y2=12.7		axilla	reference depth
100 cGy				mid‐depth
central axis			neck	exit
and midplane				reference depth
depth				mid‐depth
				exit
			upper cord	reference depth
			point	mid‐depth
				exit
			lower cord	reference depth
			point	mid‐depth
				exit
CT based	xl =5.0	med – 124°	central slice	isocenter plane
tangential	x2=4.0	lat – 308°		at isocenter
breast plan	yl =5.0		+4.0 cm slice	isocenter
CL 2100c & SCX 6 MV	y2=6.0			plane
CL 2100c & SCX 15 MV (both open fields and 30° wedge fields)			–4.0 cm slice	isocenter plane
CT based prostate	x1=4.0	180	isocenter	depth to isocenter
4 field plans CL 2100c & SCX 15 MV	x2=4.0	90		from each field
	y1=3.0	270		
(Plan #l) 180 cGy to isoceuter 25% from each field	y2=4.0	0		
(Plan #2) 180 cGy to isocenter 25% from each Field	x1=4.0	225		depth to isocenter from each field
	x2=4.0	135		
	y1=3.0	45	isocenter	
	y2=4.0	315		

Regardless of the specific case chosen, each test case should have the following characteristics:

- The case has been thoroughly reviewed for completeness and accuracy of results.
- The case is saved under a uniquely identifiable patient or plan name with the same calculation grid (0.4 cm×0.4 cm×0.4 cm voxel) as defined by the baseline result.
- The reproducibility tests include evaluation of beam MUs, point doses and parameters, isodose display (on screen and hardcopy), and text printout.

The results of the clinical case reproducibility test should be identical to the baseline case results in all respects. The only exception would be a known/expected deviation resulting from a software upgrade. Naturally, any changes due to software upgrades would require the approval of the physicist prior to clinical implementation and would establish a new set of baseline data as well.

### D. Periodicity of tests

The development of any QA program must include some assessment of benefit versus cost. The increase in the number and frequency of testing helps ensure significant error reduction, but ultimately the cost in personnel and equipment time commitment becomes prohibitive. There are several recommendations given in the literature^(^ ^1^ ^–^ ^4^ ^,^ ^9^ ^)^ concerning the frequency of tests. We chose to follow those suggested by Van Dyk et al. and presented in Table 5. The majority of tests are performed on a semi‐annual basis and when there is any change in software or hardware.

**Table 5:** TPS QA periodicities

QA test description	Periodicity
electromagnetic digitizer accuracy	quarterly
image acquisition, use, and display	quarterly semi‐annually (PMI)
hardcopy output verification	semi‐annually
monitor unit verification	semi‐annually
standard isodose verification	semi‐annually
clinical case verification	semi‐annually

## III. RESULTS AND DISCUSSION

Reproducibility tests described were implemented in the clinic in August 2002. A series of software upgrades and hardware changes has taken place since then. The software was upgraded from version 5.2g in August 2002 to versions 6.0m, 6.0s, and finally 6.2b (July 2004). The hardware was a single Ultra2 workstation through version 6.0s at which time we upgraded and expanded to two SunBlade workstations and two PinnacleMD workstations. The results of all the reproducibility tests were within the stated criteria except the hardcopy output accuracy. This is because the format of the hardcopy output was changed significantly over the course of these software upgrades. Interesting, but somewhat disturbing, was the lack of information explaining the format changes and the lack of flexibility in either developing custom hardcopy formats or choosing a format from several available templates.

A particular challenge was the development of a working relationship with the radiology department. Creating an environment for cooperation and education on the QA tests was difficult, since they have never fully understood the use of CT data in radiation therapy. The increasing use of image‐based treatment planning will require all physicists to begin educating themselves as well as the management and staff at their institution on the use of these imaging modalities in radiation therapy. Additional reproducibility tests in development include: CT‐MRI fusion, autogeneration of blocking, and autogeneration of bolus. Progression into IMRT will require the development of new tests to ensure accuracy and constancy of results from inverse planning algorithms.

## IV. CONCLUSION

The fundamental reproducibility tests presented are the foundations of any comprehensive quality assurance program for any TPS. The radiotherapy planning quality assurance program presented here (Table 1) represents a reasonable and practical program for the community setting. The fundamental tests can be expanded and/or adapted to any given radiation therapy center's particular character and needs. The complexities of modern treatment‐planning systems require a quality assurance program. While the program may be an evolutionary one, as we have outlined, certain fundamental tests must be maintained.
